# Is a Wiggling-Motion Modified Two-Step Impression Technique as Accurate as Conventional Techniques in Restorative Dentistry?

**DOI:** 10.3390/dj11050139

**Published:** 2023-05-22

**Authors:** Anastasia Zappi, Efstratios Papazoglou, Maria Anagnostou

**Affiliations:** Department of Operative Dentistry, School of Dentistry, National and Kapodistrian University of Athens, 11527 Athens, Greece

**Keywords:** dental impression technique, polyvinylsiloxane, wiggling motion technique, double mix two-step technique

## Abstract

Background: The purpose of this study was to evaluate dental impression accuracy of one-step and two-step techniques compared to a modified two-step technique. Methods: Four impression techniques were compared: (1) a one-step double mix (DM) technique, (2) a cut-out (CO) technique, in which space relief was created using a blade and a laboratory bur, (3) a membrane (ME) technique, in which space relief was created by placing a PVC membrane on top of the putty material during the primary impression, and (4) a wiggling motion (WI) technique, in which PVC membrane was placed and additional wiggling movements were performed during the first 20 s when the primary impression was seated upon the master model (MM). Impressions were poured with type IV stone. Casts were scanned with a laboratory scanner and measurements were made for each cast using three-dimensional analysis software. Results: All groups presented differences compared to MM group, in at least one intra-abutment distance. Groups DM and ME presented the most significant differences, in three and two distances, respectively, whereas CO and WI presented one significant different distance compared to MM. There were no differences between MM and the four techniques for inter-abutment distances. Conclusions: WI yielded similar results with CO technique. Both performed better than the other groups.

## 1. Introduction

Although there are difficulties associated with the measurement of quality in dentistry, quality assurance systems are based on developing mechanisms for the assessment and improvement of dental care quality [1]. An accurate dental impression is a fundamental prerequisite for manufacturing well-fitting, high-quality, indirect restorations that show good clinical behavior in the long term [2]. As far as the fabrication of single crowns and short-span fixed dental prostheses is concerned, several studies have shown that both digital and conventional impression techniques have similar levels of accuracy, leading to restorations with marginal discrepancies and internal fit within the clinically acceptable limits [3,4]. However, digital impression techniques are not yet so widely used due to high costs of purchasing the necessary equipment [5]. On the other hand, conventional impression techniques with elastomeric materials remain the most frequently performed procedures [6,7].

Among the broad range of impression materials, vinyl polysiloxanes (VPS) or addition-type silicones are the most commonly used material in fixed prosthodontics [6,8,9,10]. They are available in four different consistencies according to ISO 4823:2015 standard (type 0, putty; type 1, heavy body; type 2, medium body; and type 3, light body), which can be used in combination (two-step) or solely (one-step). Usually, two-phase impressions are made with putty or heavy body material together with light body/wash material using stock trays, whereas one-phase impressions are conducted with medium body material and custom trays. According to in vitro studies, the one-phase technique seems to be inferior, since casts originating from one-phase impressions were found to be less accurate [11,12,13,14,15].

The two-phase DM impression technique can be conducted in a one- or two-step procedure, with the latter having the disadvantage of increased chairside time. When the one-step DM technique is chosen, materials of different viscosities are simultaneously mixed by two different operators. Wash material is applied on prepared tooth surfaces, while putty material is placed on the tray and then inserted simultaneously in the mouth. In the two-step procedure, a preliminary impression with putty must be taken (first step) and only after the complete setting of putty is wash material applied and preliminary impression reinserted (second step). There is some controversy, but the majority of studies have reported that two-step techniques are more accurate than one-step, which may be attributed to the uncontrolled bulk of wash material in one-step technique [11,12,13,15,16,17,18,19].

In two-step DM techniques, there is the need for creating space relief in the preliminary impression to accommodate the wash material. A well-known procedure is the CO technique, where vents and/or grooves are created in preliminary impression and relief space is prepared with the use of scalpel, sharp instruments, or rotary burs [20,21]. The major drawbacks of the CO technique are that it is time-consuming and that a uniform space for wash material is not easy to achieve. Alternative ways for creating space relief have been described as placing spacer copings or membrane prior to taking the preliminary impression [12,20,21,22,23]. It was reported that the desired wash material thickness in two-step impressions should be 1–2mm, since it resulted in the highest accuracy according to several studies [22,24,25].

Despite spacer copings of certain thickness giving very accurate casts, their fabrication requires an additional laboratory step. On the other hand, the use of membrane is easy and simple, but it may result in thin relief space for wash material. The authors suggest a modified technique for taking two-step putty/wash dental impressions using plastic wrap and performing a horizontal wiggling motion during the first 20s of setting of the preliminary impression, in order to increase the space for wash material.

The objective of this study was to evaluate the dimensional accuracy of single-crown and three-unit fixed partial denture casts that originated from four different putty/wash impression techniques, including a one-step putty/wash technique, a two-step CO technique, a two-step ME technique, and a two-step technique using membrane and wiggling motion. The null hypothesis was that no differences in dimensional accuracy exist between MM and stone casts obtained from the four impression techniques.

## 2. Materials and Methods

**Figure 1:**
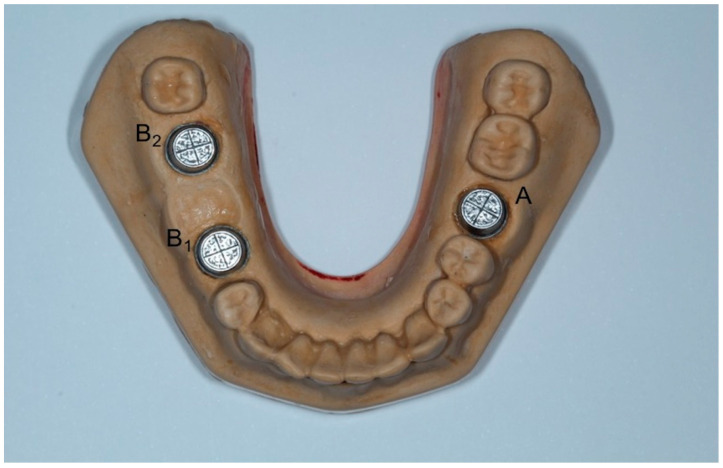
Master model with three metal dies (A, Β_1_, Β_2_), representing one full-crown preparation for a single crown (first molar-A) and two prepared teeth for a three-unit FPD (right second premolar-B_1_ and second molar-B_2_) at the opposite site.

**Figure 2:**
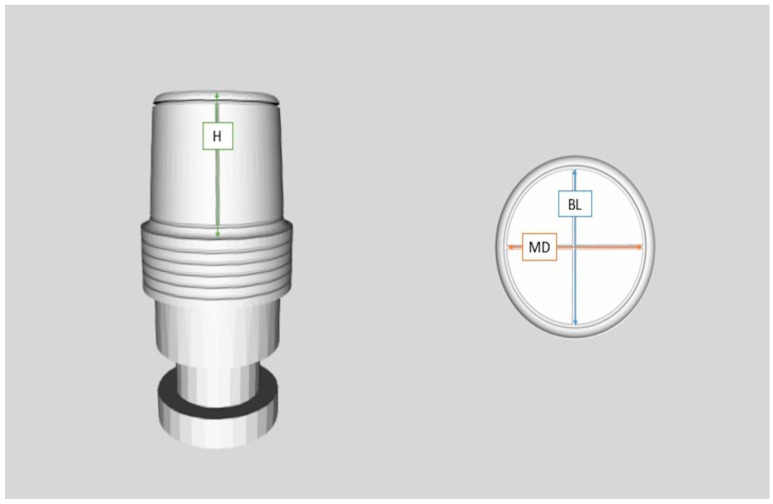
Design of metal dies, where grooves that represent height (H) (left), mesiodistal (MD), and buccolingual (BL) distances (right) are highlighted.

**Figure 3:**
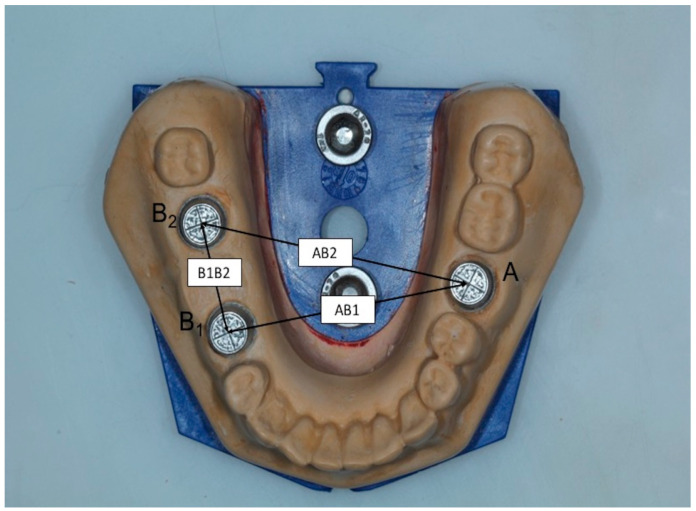
The point where BL and MD grooves met was referred to as the center of each die (point A, B_1_, B_2_). Three inter-abutment measurements (AB1, AB2, B1B2) were obtained, using the center of each metal die as reference point.

A master model was made which incorporated three metal dies, representing one full-crown preparation for a single crown (first molar-A) and two prepared teeth for a three-unit FPD (right second premolar-B1 and second molar-B2) at the opposite site. The MM was replica of a lower jaw Typodont model made of type IV gypsum (Fujirock EP, GC Europe, Leuven, Belgium) which had been modified to simulate the above-mentioned clinical situation (Figure 1). The metal dies were designed using three-dimensional analysis software Rhino 6 (Rhinoceros, McNeel Europe, Barcelona, Spain) (height: 6.00 mm; occlusal diameter: 6.69 mm; cervical diameter: 7.42 mm) and were fabricated using laser sintering technology and then fixed in place with type IV gypsum. A circular groove at the periphery and two vertical grooves were fabricated at the top of each die representing buccolingual (BL) and mesiodistal (MD) dimensions, whereas a longitudinal groove at the buccal side was formed to represent height (H) (Figure 2). The point where BL and MD grooves met was referred as the center of each die (point A, B_1_, B_2_) (Figure 3).

**Figure 4:**
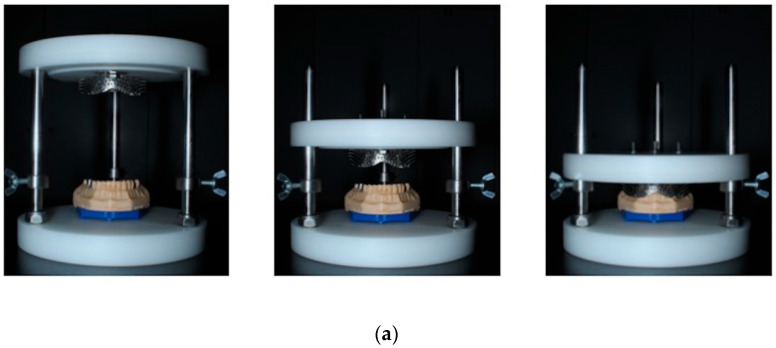

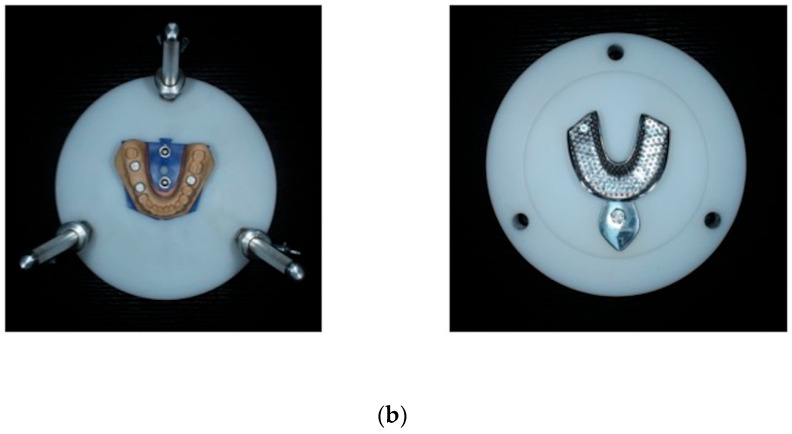
A custom-made apparatus was used in order to achieve repeatable positioning of the tray. (**a**). Front view and (**b**). Top view of the apparatus, the tray, and the master model.

The MM served as a reference and multiple impressions were taken from it (*n* = 10 for each group, for a total of 40). Metal perforated stock trays with rim-lock design (Asa Dental size 6, Bozzano, Italy) were used to take the impressions. In order to achieve repeatable positioning of the tray, a custom-made apparatus was designed aiming to standardize the impression conditions (Figure 4). Addition silicone was used in putty and wash consistency (DM technique) (Express STD, and Express 2 XT Light Body, 3M ESPE, St. Paul, MN, USA) with no tray adhesive. Due to the fact that impressions were taken at room temperature and not intraorally, the setting time was doubled, from the manufacturer’s instructions for intraoral use, to 9 min.

The impressions were taken using four different techniques (groups) and each group consisted of ten impressions/casts. The first group was obtained using a one-step technique, whereas the other three were obtained using two-step techniques. For the one-step DM technique group, putty and lightweight silicone impression materials were simultaneously mixed by two different operators. Putty was placed in the tray, while lightweight material was dispensed through an automix cartridge directly to the metal dies of the master model. The tray with the putty material was then pressed over the MM using the positioning appliance, and the impression was taken in one step.

For the groups where double-step techniques were used, a primary impression with putty material was taken, and then the definite impression was obtained by placing only lightweight material and a reseating of the primary impression. For the CO group, the space relief for the lightweight material was created using a No.11 blade and rotary laboratory bur. The procedure of creating space relief was performed by the same operator for all impressions of the CO group and lasted approximately 2 min for each impression. For the ME group, the space relief was created by placing a piece of 20 μm thickness PVC membrane (Sanitas cling film, Sarantis SA, Athens, Greece) on top of the putty material, and the initial impression was taken with that in place, interfering between putty material and MM. Finally, for the WI group, PVC membrane was also used, but in addition to that, a wiggling motion was performed when the primary impression was seated upon the MM for the first 20 s, in order to create the relief space. More specifically, horizontal circular wiggling motion of 2–3 mm was performed after adapting the primary impression upon the MM. After that, in contrast to the other groups, the primary impression was taken off and left undisturbed until setting time. In WI group, the appliance for the standardized positioning of the tray was not used in the first phase of the putty impression.

All impressions were stored at room temperature for at least 1 h before pouring (21 °C room temperature, 55% humidity). The impressions were sprayed once with dental surfactant (Smoothex Debubbling Solution, Whip Mix Europe GmbH, Dortmund, Germany) and then poured in type IV dental gypsum (Fujirock EP, GC Europe, Leuven, Belgium). A ratio of 20 mL distilled water and 100 g dental gypsum was used as recommended by the manufacturer. The gypsum was manually mixed during the first 15 s and then mechanically mixed under vacuum for 45 s. Next, the mixture was poured into the impressions, mechanically vibrated until they were totally filled and allowed to set for more than 40 min before being separated from the impressions (Vibr-X-24, OMEC, Muggio, Italy). The procedure was performed by a single operator.

All stone models as well as the MM were scanned by a laboratory scanner (Aadva Lab Scan, GC Tech, Breckerfeld, Germany) with nominal accuracy <10 μm. Metal pieces of MM were covered with anti-reflective spray (Helling 3D Laser Scanning Spray, Helling GmbH Heidgraben, Germany). The measurements were made from the scans using the three-dimensional analysis software Rhino 6 with absolute nominal accuracy at 10 μm. Three intra-abutment measurements (MD, BL, and H) were obtained for each metal die (A, B1, B2) and the mean values of the three metal dies were calculated. Three inter-abutment measurements (AB1, AB2, B1B2) were also obtained, using the center of each metal die as reference point (Figure 3). Overall, twelve distances were measured at each cast, including three intra-abutment distances for each of the three metal dies (a total of nine measurements) and three more inter-abutment distances. The intra-abutment distances of the dies were grouped together, resulting in three intra-abutment distances at each cast, which referred to all three dies. Each distance was measured three times and the mean value was calculated. MM was considered as control group. All measurements were performed by one examiner.

Statistical analysis was performed with SPSS 25.0 for Windows (ΙΒΜ, Armonk, NY, USA). Databases were tested for normality using the Shapiro–Wilk test. When the assumption of normality was met at all groups, analysis of variance (ANOVA) was used for investigating statistically significant differences between groups. If there were statistically significant results, repeated measures were done using the Bonferroni method. By contrast, when the assumption of normality was not met at all groups, the nonparametric Kruskal–Wallis test was used for investigating statistically significant differences between groups. If there were statistically significant results, the Bonferroni approach was followed to identify the pairs of groups in which significant differences were found. All statistical tests were two-tailed, with the level of significance set at *p* < 0.05.

## 3. Results

Table 1 and Table 2 summarize the results. For all groups, intra-abutment distances were found to be statistically different from the MM group at least in one distance. MD distances in all groups were significantly larger than the MM group (*p* < 0.001), but no statistically significant differences were observed among the rest of the groups. In DM and ME groups, BL distances were significantly larger than in the MM group (*p* < 0.001), but no statistically significant differences were observed between CO and MM or WI and MM. There were no statistically significant differences among test groups for BL distances. Finally, the H values of dies in the DM group were statistically significantly smaller than in the MM group (*p* = 0.043), but no statistically significant differences were observed among the tested groups.

As far as the inter-abutment distances are concerned, statistically significant differences were found only in the AB2 distance. More specifically, the AB2 distance in WI group was found to be statistically different than in the group DM (*p* = 0.028), but no significant differences were observed between any other group and MM.

## 4. Discussion

In the present study, the dimensional accuracy of casts that originated from four different putty/wash impression techniques was investigated. The null hypothesis was rejected. According to our findings, two-step techniques performed better than the one-step technique, and among them, CO and WI had the best results.

Several methods have been described in order to assess dimensional accuracy. Firstly, a traveling microscope was used [26,27], followed by a toolmakers microscope that was attached to two data processors [28,29]. Certain studies used a vertical profile projector [14,16,17,18,19,20], while in others, three-dimensional coordinate measurement machines were preferred [21,30]. Another measuring method that has been described is via image measuring software. Images were taken through a digital camera [31], or a digital camera attached to a microscope [32,33], and then measurements were taken through calibrated image software. For the present investigation, we used a laboratory scanner to scan all the models, and then STL files were processed through three-dimensional analysis software to produce all the measurements. The simultaneous absolute horizontal arrangement of three abutments on the same cast, with possibly different long axes, is practically impossible. Thus, in a certain degree, the projected areas of the abutments can be measured, which may impose a limitation to the measurement accuracy. However, this applies to all measurements, rendering this limitation less influential. On the other hand, according to the technical specifications of the scanner, the measurement accuracy is 4 μm; consequently, the measurements of the projected surfaces, which are in the range of 6 mm to 4.5 cm, are considered precise. Similar protocol, with a laser scanner instead, was also used by Dugal et al. [17].

In our study, a solid custom-made MM was fabricated, which consisted of a gypsum cast and three machined metal dies. The dies were rigidly fixed in place with gypsum to avoid any source of error due to dies’ mobility. This was favored instead of the use of a prefabricated acrylic dentoform cast and acrylic teeth fixed with resin, as, in the study of Singh et al. [20], there was referred a risk of micro movements after multiple impressions. The dies in our study represented full-crown preparations and were machined according to ADA specifications, but shorter in height. ADA specifications were also followed by other investigators [14,34,35], or in similar designs with different taper [28,30], and undercuts [32] were also utilized. The use of rectangular-shaped dies [19,29] has also been described, but their form is far from any clinical situation, so they were not chosen in our study.

The majority of in vitro studies that investigate impression accuracy used one to three metal dies fixed in a metal base as master models [14,21,31,34]. Even though, in some studies, varying undercuts were made at the cervical part of metal dies [26,32], those simplified master models cannot safely simulate clinical conditions. In order to get clinically relevant results, we tried to recreate a common clinical situation by using three metal dies in a partial edentulous arch, similar to two other studies [21,33]. The dies simulated full-crown preparations, two for a FPD and one for a single crown at the opposite site. Intracoronal markings and a missing tooth gave us the ability to measure intra- and inter-abutment distances in a more realistic set up, in order to evaluate the dimensional accuracy of different impression techniques. A custom-made apparatus was designed and fabricated to standardize the impression conditions during positioning of the tray, so that we could omit errors caused by different handling procedures. The only difference between the WI technique and the CO and DM techniques was the way the space for the wash material was created. When the wash material was added in the preliminary impression of WI, the tray was reseated using the same apparatus, as with the other two techniques. Consequently, this does not differentiate the standardization of the various techniques.

Most intra-abutment distances were found to be equal or larger than the master model’s, which was also observed in some other studies [14,19]. Only the H of dies in group DM was found to be smaller than the MM, whereas inter-abutment distances did not differ statistically in any group from MM. Nissan et al. also found decreased height of dies but increased inter-abutment distances [16]. On the other hand, Idris et al. and Mann et al. found increased inter-abutment distances but decreased intra-abutment distances only in CO group [21,28], which contradicts with the results of our study.

According to our results, the diameter of the dies was found to be increased in comparison to the MM in MD and, in some groups, in the BL direction too. From a clinical point of view, this would not have impaired the seating of the restoration, since more space for the cement would have been created. The maximum dimension difference was at the range of 50 μm, which is in the acceptable fitting range. Considering that the cement thickness would increase by 50 μm, this could not be considered critical for the retention and resistance of a prosthesis. On the contrary, the H of the dies in group DM was found to be smaller than the MM. Shorter dies would negatively affect the marginal adaptation of the restoration. This discrepancy in height could not be predictably regulated by the application of die spacer prior to fabrication of the restoration.

Putty/wash impressions can be conducted using either one-step or two-step techniques. Some authors have suggested that the impression technique does not play a significant role in the accuracy of impressions with addition-type silicone [23,31,33]. On the contrary, there are many studies which suggest that two-step techniques result in more accurate casts than the one-step technique [14,15,19]. The same conclusion has been drawn evaluating our results. The impression techniques that were used in the present study were a one-step DM technique, a two-step CO technique, a two-step ME technique, and a two-step WI technique. Groups CO and WI were found to be more accurate, since they had the least statistically significant differences compared to MM, whereas DM was found to be the least accurate.

Among the different two-step techniques, Mann et al. found that the membrane technique was more accurate than the cut-out technique, but there was a higher risk of an incompletely reproduced preparation margin [21]. That finding does not agree with our study, in which the CO technique had less statistically significant differences than the ME technique from MM. Those differences in results may be attributed to different experimental set-ups, since Mann et al. used a Plicafol (GS Folienfertigung, Lebach, Germany) membrane and the only intra-abutment measurements done were the diameters of dies.

Pastoret et al. used a similar technique, by the name “separating foil technique”. They used Plicafol as a separating membrane and performed horizontal movements every 2 s for 5 min until the preliminary impression was taken off. Another difference from our study is that they used regular body as wash material for the final impression and epoxy resin as cast material. Their study found no differences between one-step and separating foil techniques in intra-abutment and inter-abutment distances [33]. An in vivo study by Silva et al. also evaluated a similar technique with horizontal movements but without the use of membrane and found no significant differences between the investigated techniques, which were a one-step technique, a membrane technique, a cut-out technique, and a two-step technique without space relief [31]. That finding should not be surprising, since it is possible that controlled in vitro conditions may exacerbate the differences in accuracy between techniques that are not detected in clinical conditions. Contrary to Pastoret et al. and Silva et al. who used similar techniques to WI group, we chose to use common PVC membrane instead of Plicafol, since it is readily available in every dental practice. In addition, in our experimental set-up, the preliminary impression was taken off the MM after 20 s and left undisturbed until final setting. In clinical conditions, it corresponds to reduced intraoral setting time for preliminary impression, making it more comfortable for the patient. Despite those two modifications regarding previous studies, the WI technique was as accurate as the CO technique and more accurate than the other two groups. The fact that WI and CO techniques created bigger space relief for wash material may be the reason for their better performance.

A limitation of the current investigation was the lack of use of any disinfection method. Many in vitro studies have evaluated the effect of disinfection procedure on dimensional accuracy of elastomeric impression materials [36,37,38]. Pal et al. reported that all the disinfectants studied produced complete disinfection, and simultaneously did not cause any deterioration in surface detail reproduction of the casts [38]. Additionally, the dimensional changes of polyether and vinyl polysiloxane impression materials immersed in different disinfectants have been studied. According to Soganci et al. [37], there was no significant difference in dimensional accuracy between the two elastomeric impression materials tested. Both materials showed similar dimensional accuracy and excellent stability. Most of the research studies have shown that different disinfectant solutions and storage times have a different effect on the impression materials; however, the dimensional changes are usually minor and clinically acceptable [39,40,41]. It was assumed that, since we used the same impression materials in all groups, disinfection means would affect all groups in the same way and they would not alter the results. Future research could be conducted comparing the accuracy of different categories of impression materials or patient satisfaction of these impression techniques.

In conclusion, within the limitations of this study, it was found that a modified wiggling motion technique yielded similar results to the cut-out technique, and both performed better than the one-step double mix and membrane techniques.

## Figures and Tables

**Table 1:** Median values of intra-abutment distances per group (interquartile range is presented into brackets). Distances marked with the same letter in a row had no statistically significant differences [one-step technique (DM), cut-out technique group (CO), membrane group (ME), wiggling motion group (WI), master model (MM)] (*n* = 10, *p* < 0.05).

Distance (μm)	DM	CO	ME	WI	MM
MD	6064 ^a^ (59.49)	6067 ^a^ (84.75)	6080 ^a^ (44.59)	6070 ^a^ (27.34)	6029 ^b^ (37.50)
BL	6073 ^a^ (55.00)	6049 ^ab^ (81.58)	6068 ^a^ (67.25)	6042 ^ab^ (37.00)	6026 ^b^ (20.84)
H	6017 ^b^ (95.58)	6001 ^ab^ (72.67)	6029 ^ab^ (78.83)	6023 ^ab^ (72.92)	6052 ^a^ (44.67)

**Table 2:** Mean values of inter-abutment distance per group (standard deviation is presented into brackets). Distances marked with the same letter in a row had no statistically significant differences [one-step technique (DM), cut-out technique group (CO), membrane group (ME), wiggling motion group (WI), master model (MM)] (*n* = 10, *p* < 0.05).

Distance (μm)	DM	CO	ME	WI	MM
AB_1_	40,109 ^a^ (41.02)	40,104 ^a^ (69.79)	40,122 ^a^ (64.62)	40,108 ^a^ (52.98)	40,046 ^a^ (54.43)
AB_2_	44,676 ^a^ (29.88)	44,718 ^ab^ (36.06)	44,734 ^ab^ (69.10)	44,748 ^b^ (52.60)	44,720 ^ab^ (48.20)
B_1_B_2_	16,847 ^a^ (57.00)	16,861 ^a^ (71.06)	16,857 ^a^ (91.49)	16,795 ^a^ (50.77)	16,806 ^a^ (64.56)

## Data Availability

Data is contained within the article.
